# The Cellular Composition of the Innate and Adaptive Immune System Is Changed in Blood in Response to Long-Term Swimming Training

**DOI:** 10.3389/fphys.2020.00471

**Published:** 2020-05-12

**Authors:** José P. Morgado, Catarina N. Matias, Joana Filipa Reis, Dalia Curto, Francisco Bessone Alves, Cristina P. Monteiro

**Affiliations:** ^1^Laboratory of Physiology and Biochemistry of Exercise, Faculdade de Motricidade Humana, Universidade de Lisboa, Lisbon, Portugal; ^2^Instituto Superior de Ciências Educativas, Lisbon, Portugal; ^3^Universidade Europeia, Lisbon, Portugal; ^4^Interdisciplinary Center for the Study of Human Performance (CIPER), Faculdade de Motricidade Humana, Universidade de Lisboa, Lisbon, Portugal

**Keywords:** training season, swimmers, leukocytes, lymphocytes subsets, upper respiratory symptoms

## Abstract

Competitive swimming requires high training load cycles including consecutive sessions with little recovery in between which may contribute to the onset of fatigue and eventually illness. We aimed to investigate immune changes over a 7-month swimming season. Fifty-four national and international level swimmers (25 females, 29 males), ranging from 13 to 20 years of age, were evaluated at rest at: M1 (beginning of the season), M2 (after the 1st macrocycle’s main competition), M3 (highest training load phase of the 2nd macrocycle) and M4 (after the 2nd macrocycle’s main competition) and grouped according to sex, competitive age-groups, or pubertal Tanner stages. Hemogram and the lymphocytes subsets were assessed by automatic cell counting and by flow cytometry, respectively. Self-reported Upper Respiratory Symptoms (URS) and training load were quantified. Although the values remained within the normal range reference, at M2, CD8^+^ decreased (M1 = 703 ± 245 vs. M2 = 665 ± 278 cell μL^−1^; *p* = 0.032) and total lymphocytes (TL, M1 = 2831 ± 734 vs. M2 = 2417 ± 714 cell μL^−1^; *p* = 0.007), CD3^+^ (M1 = 1974 ± 581 vs. M2 = 1672 ± 603 cell μL^−1^; *p* = 0.003), and CD4^+^ (M1 = 1102 ± 353 vs. M2 = 929 ± 329 cell μL^−1^; *p* = 0.002) decreased in youth. At M3, CD8^+^ remained below baseline (M3 = 622 ± 245 cell μL^−1^; *p* = 0.008), eosinophils (M1 = 0.30 ± 0.04 vs. M3 = 0.25 ± 0.03 10^9^ L^–1^; *p* = 0.003) and CD16^+^56^+^ (M1 = 403 ± 184 vs. M3 = 339 ± 135 cell μL^−1^; *p* = 0.019) decreased, and TL, CD3^+^, and CD4^+^ recovered in youth. At M4, CD19^+^ were elevated (M1 = 403 ± 170 vs. M4 = 473 ± 151 cell μL^−1^; *p* = 0.022), CD16^+^56^+^ continued to decrease (M4 = 284 ± 131 cell μL^−1^; *p* < 0.001), eosinophils remained below baseline (M4 = 0.29 ± 0.05 10^9^ L^–1^; *p* = 0.002) and CD8^+^ recovered; monocytes were also decreased in male seniors (M1 = 0.77 ± 0.22 vs. M4 = 0.57 ± 0.16 10^9^ L^–1^; *p* = 0.031). The heaviest training load and higher frequency of URS episodes happened at M3. The swimming season induced a cumulative effect toward a decrease of the number of innate immune cells, while acquired immunity appeared to be more affected at the most intense period, recovering after tapering. Younger athletes were more susceptible at the beginning of the training season than older ones.

## Introduction

It is generally acknowledged that the immune system may experience a functional reduction when exposed to successive psychological and physical stressful stimulus, such as the competitive training process (Walsh et al., 2011). In order to attain high level performances, endurance athletes, such as swimmers, undertake large periods of intense training with short recovery periods (Aubry et al., 2014). These training conditions aim to stimulate adaptive mechanisms related to metabolic, hormonal, circulatory and respiratory responses to improve performance. However, if a transient imbalance between training loads and recovery occurs, it may contribute to the onset of fatigue, compromise attendance to training and performance and negatively influence health status, although these effects may be reversible by a tapering or recovering period (Mujika et al., 2000; Mujika, 2010). If subsequent extra pressure is generated, a state characterized by substrate depletion and hormonal and immune functions disturbances may arise, from which athletes often report infection episodes after hard training periods (Gleeson, 2007; Cordova et al., 2010; Dias et al., 2011; Morgado et al., 2012; Gleeson and Williams, 2013; Rama et al., 2013).

Longitudinal studies that followed up a 7-month swimming training season reported a reduction in neutrophils and monocytes resting values (Morgado et al., 2012) and decreased CD56^+^ NK cells (Gleeson et al., 1995; Rama et al., 2013). After a 3-month swimming training program CD56^+^ NK cells were also diminished (Gleeson et al., 2000). So, it seems that long-term intensified training can affect the number of circulatory innate immune cells.

However, the magnitude of the decreases observed is small and frequently the values are kept inside the interval of normal reference values, not anticipating immune depression. In addition, a reduction in the numbers of immune cells does not necessarily implicate immunosuppression, and instead is most likely an immune-surveillance response (Campbell and Turner, 2018). Nevertheless, frequently athletes report high incidence of upper respiratory infection (Walsh, 2018). So, it is reasonable to ask which immune variables are relevant to monitor and control to help coaches and athletes to hand the transitory impaired performance and eventual immune perturbation state that is frequently reported in periods of heavy training. The answer to this question may be crucial for adequate periodization of training, and eventually to the individualization of the training process.

Additionally, T and B cells have shown sign of hampered functionality in athletes engaging long-term periods of intense training (Walsh et al., 2011).

To our knowledge, no one has addressed the effect of a long-term training process of any physical activity or sports controlling for participants characteristics on the circulating leukocytes and subpopulations (including lymphocyte subset populations). Thus, this study aimed to investigate the changes of resting systemic immunological cell parameters over the course of a 7-month swimming training season, in well-trained swimmers, taking into account sex, maturity, and age.

We hypothesized that the most intense periods of training over the competitive training season would lead to some immunological perturbation, and that the URS occurrence would be greater during this period. Conversely, by the end of the training season, where recovery is provided and peak performance expected, the immune system would recover thus conferring an healthy condition necessary for achieving best performances in competition (Aubry et al., 2014).

## Materials and Methods

### Participants

Fifty-four national and international level swimmers (25 females, 29 males), with a competitive swimming background of 5.5 ± 0.3 mean years, ranging from 3 (youth) to 11 (seniors) years of competitive practice, undertaking 13–15 h of pool training and 4 h of dry-land training per week, were evaluated in this study. The swimmers were divided into three swimming age groups according to the regulation of the *Portuguese National Federation* and the *Ligue Européene de Natation* (*LEN*) (youth: *n* = 29, 13–14 years in females and 14–15 years in males; juniors: *n* = 13, 15–16 years in females and 16–17 years in males; seniors: *n* = 12, ≥17 years in females and ≥18 years in males) or into different maturity groups (late pubertal: *n* = 34; mature: *n* = 20).

After receiving detailed information about the aim of the study and the possible risks of the investigation, either the swimmers or their parents, as appropriate, provided their written informed consent to participate. All procedures were approved by the Ethics Committee of the Faculty of Human Kinetics of the University of Lisbon and were conducted in accordance with the Declaration of Helsinki for human studies (World Medical Association, 2008). During the period of observation athletes were asked not to take dietary supplements, nor any kind of medication other than that prescribed for episodes of acute illness.

### Study Design

This study used an observational design with a follow-up over a swimming training season lasting 30 weeks. Swimmers followed the training program set by their coaches. Participants included two youth training groups each trained by different coaches and two training groups assembling juniors and seniors each trained by the head coach of the respective squad. The evaluation of the swimmers was made at rest at four assessment points: M1 (at the beginning of the season; baseline evaluation), M2 (after the main competition of the 1st macrocycle; 13th week of training), M3 (week with the highest training load of the 2nd macrocycle, 23rd week of training) and M4 (after the main competition of the 2nd macrocycle; 30th week of training). At each assessment point, data collected for all participants included body composition, biological maturity (pubertal Tanner stages) and biochemical immune indices. Athletes were instructed not to consume anything but water after 10 p.m. preceding the day of evaluation and to have a minimum of 8 h rest before testing. The body composition measurements and the resting blood sample collection were performed in a fasted state (between 6:00 and 7:00 a.m.). Throughout the training season the incidence of self-reported illness symptoms and the menstrual cycle phases for girls were monitored weekly and training load of all scheduled swimming sessions was quantified. The characteristics of the training regimens and competition schedules were not modified by the present study in anyway.

### Body Composition Measurements

Height and body mass (BM) were measured in the fasted state wearing a bathing suit without shoes. Stature was measured to the nearest 0.1 cm (Siber-Hegner anthropometric kit). BM and Fat mass percentage (%FM) were assessed using Bioelectrical Impedance Analysis (TANITA BC-601 body composition scale monitor) with a measuring current of 50 kHz, 100 μA. Fat mass (FM) was calculated according to the formula: FM (kg) = BM × %FM/100. Free fat mass (FFM) was calculated according to the formula: FFM (kg) = BM – (BM × %FM/100).

### Maturity – Tanner Stages

Pubertal stage was assessed as described elsewhere (Morgado et al., 2018). As a consequence of the heterogeneity in chronological age of the swimmers, at M2, some swimmers classified themselves as having developed into the subsequent stage in comparison to M1. Yet, all swimmers maintained the classification they had at M2 throughout the rest of the training season (M3 and M4). So, in this study, we chose to classify the swimmers according to their maturity stage at M2. As all swimmers classified themselves as late pubertal or mature, only two maturity groups were established.

### Swimming Training Season

The study was divided into three main periods that represented distinctive training phases of a 7-month swimming season: M1 to M2 (3 months) corresponded to the 1st macrocycle of the training season. This period was characterized by an aerobic training predominance and the progressive increase of training volumes and intensities in the first 2 months and for the maintenance of high intensities and progressive decrease of volumes in the last month; from M2 to M3 (2 months) the training was characterized by a progressive increase in training volume, intensity and frequency. When the peak of the training load of the season was reached, the swimmers were evaluated (M3). In this period there was also a more frequent participation in preparatory competitions; from M3 to M4 (1 month) the training load was progressively reduced in order to obtain peaking at the main competition of the macrocycle. Athletes were evaluated just after the main competition of the season (M4).

### Quantification of the Training Load

The training load of each session was assessed by quantifying the volume (total amount of meters swum), the weighed volume (sum of the meters swum in each zone of intensity, multiplyed by the respective index), and arbitrary units of load (AUL) based on previous investigations (Mujika et al., 1995, 1996; Morgado et al., 2012, 2018; Rama et al., 2013). The weekly load was characterized by the sum of the load of all the training sessions of the week and the comparison between the assessment points was performed based on the mean of the training load of the 4 weeks prior to each assessment point.

### Immune System Parameters

Peripheral venous blood samples were collected via standard procedures between 6:00 and 7:00 a.m., in the fasted state, at least 12 h after the last training session, at the four assessment points (M1 25.6 ± 11.4 h, M2 24.4 ± 11.0 h, M3 23.6 ± 12.8 h, and M4 23.6 ± 11.8 h; range 12–40 h at any assessment point, *F* = 0.529; *p* = 0.612). Venous blood was collected into tubes containing EDTA for assessment of hemogram and leukogram and for counting of total lymphocytes (TL) and subsets (CD3^+^, total T lymphocytes cells; CD4^+^, T helper cells; CD8^+^, T cytotoxic cells, CD16^+^56^+^, Natural Killer (NK) cells; and CD19^+^, B cells). Hemogram and leukogram were performed in an automated hematology analyzer (Coulter LH 750, Beckman) which produced information about the following parameters: hemoglobin concentration (g dL^–1^), hematocrit (%); and counts of white blood cells, namely, leukocytes, neutrophils, monocytes and eosinophils. Total lymphocytes and subsets were counted by flow cytometry (FACS Calibur, BD Biosciences), using the commercial kits from BD Biosciences (BD multitest IMK kit). Results were expressed as number of cells 10^9^ L^–1^ for leukogram parameters and as number of cells μL^−1^ for total lymphocytes and subsets. Plasma volume variation was calculated between the assessment points according with the methods of Dill and Costill (1974).

### Upper Respiratory Symptoms

Upper respiratory symptoms were self-reported by the athletes using daily log books as fully described in our previous work (Morgado et al., 2018). The symptoms registered were headache, fever, ear pain, chills, runny or blocked nose, pharyngitis/tonsillitis, bronquitis, asthma, phlegm, cough, conjunctivitis, itchy, watery eyes, nausea/vomiting and diarrhea. As described by Bishop (2006), if more than two symptoms were repeated on at least two consecutive days, one episode was defined. A new episode was considered after a minimum interval of 10 days following the previous one. Additionally, all swimmers were asked to indicate the medication they were on, in case of an episode occurrence.

### Statistical Analysis

The statistical analyses were performed using the software IBM SPSS Statistics (version 21; IBM Corp., Armonk, NY, United States) and the R software (version 2.15.1; R Core Team), both for Windows, with a significance level of 5%. Descriptive statistics, including means and standard deviation (mean ± SD) were performed for all outcome measurements. To verify if participants were within the clinically normal reference range values associated with each variable, the one sample *t* test was used to compare group means with the upper or lower limits of the reference interval (Lewis et al., 2006; INSA, 2011).

We evaluated whether sex, maturity, and swimming age group influenced the effect of training on the immune response by using non-parametric mixed-design ANOVAs. The within-subjects factor was the assessment points (four levels: M1, M2, M3, and M4), which is referred to as the effect of training, and the subjects’ factors were the aforementioned influential variables sex, maturity, and swimming age group. The non-parametric mixed-design ANOVA has an ANOVA-type statistic (ATS) for each effect, and also a modified ANOVA-type statistic (MATS) for the subject’s factor. The option for the non-parametric approach was due to the violation of the assumptions of parametric mixed ANOVA, namely the normality of the dependent variables in each factor’s level, homogeneity of variances or sphericity. This non-parametric analysis was performed with the *nparLD* package (Noguchi et al., 2012) from the R software. Subsequent analyses were performed according to procedures adopted previously (Morgado et al., 2016, 2018).

Repeated measures ANOVA was used for the assessment of training effects on immune parameters. Normality and sphericity assumptions were evaluated with the *Shapiro-Wilk* and *Mauchly’s* test, respectively. *Post hoc* tests with *Bonferroni* correction were performed to determine between which assessment points a significant difference was observed. If the repeated measures ANOVA assumptions were not met, the exercise effect was assessed by *Friedman* test. *Post hoc* analyses were performed using *Dunn-Bonferroni* test (Dunn, 1964) or, if necessary, due to the conservative characteristic of the *Bonferroni* procedure, according to Conover (1999).

Whenever there was a significant interaction between the effect of sex, swimming age groups or maturity and the effect of training on the immune parameters the subsequent analyses were performed separately for each group. Results presented for each immune cell subpopulation show only the significant interactions or differences between groups. Separate graphs along with multiple lines are presented when more than one characteristic influenced the effect of training on the immune parameters, and multiple lines in one graph when only one characteristic influenced the effect of training or when there was a difference between groups independent of training effect.

## Results

The participants’ characteristics, including demographics and body composition related variables, are presented in Table 1. Swimmers’ physical characteristics changed over the season, especially between M1 and M3. These alterations reflect height growth between M1 and M2 in males and between M2 and M3 in both groups, and also increases in BM and FFM. No swimmer suffered from major injury or sickness preventing them from training for two or more consecutive days.

**TABLE 1:** Mean ± SD values of the demographics and body composition of female (*n* = 25) and male (*n* = 29) swimmers at the four assessment points (M1, M2, M3, and M4).

Swimmers characteristics	Females				Males			
	M1	M2	M3	M4	M1	M2	M3	M4
Stature (cm)	163.0 ± 6.3***	163.5 ± 6.2	164.2 ± 6.2**	164.5 ± 6.1*;**	172.1 ± 7.5***	173.0 ± 7.0*	173.4 ± 6.9**	173.7 ± 6.7*;**
Body mass (kg)	54.9 ± 7.4***	55.5 ± 7.2	56.2 ± 7.2**	56.3 ± 6.9*;**	64.0 ± 8.0***	64.2 ± 8.0	65.4 ± 7.7**	65.4 ± 7.2**
FM (%)	23.9 ± 3.6	24.9 ± 3.5*	24.2 ± 3.5	24.2 ± 3.6	16.1 ± 3.1***	16.5 ± 3.2	16.4 ± 3.0**	16.4 ± 3.1*
FFM (kg)	41.7 ± 5.0	41.6 ± 5.0	42.5 ± 5.1**	42.6 ± 4.8**	53.6 ± 6.6	53.6 ± 7.1	54.7 ± 7.0	54.7 ± 6.3

FM, fat mass percentage; FFM, fat-free mass; * Different from M1; ** Different from M2; *** Different from M3 (p < 0.05).

The mean values of the panel of immune markers evaluated were within the reference interval associated with each variable at the four assessment points (Lewis et al., 2006). Training influenced plasma volume values at rest (*F* = 7.446, *p* = 0.000) with a decrease from M1 to M2 (59.9 ± 3.1 vs. 58.6 ± 3.2%; D = −1.3%; *p* < 0.001), and increases from M2 to M3 (58.6 ± 3.2 vs. 59.4 ± 3.3%; D = 0.8%; *p* = 0.021) and to M4 (58.6 ± 3.2 vs. 59.6 ± 3.3%; D = −1.0%; *p* = 0.002). Plasma volume variation according to Dill and Costill (1974) from M1 to M2 was −7.02 ± 7.51% (*t* = −6.868, *p* < 0.001), from M1 to M3 was −2.16 ± 9.27% (*t* = −1.709, *p* = 0.093) and from M1 to M4 was −2.42 ± 6.99% (*t* = −2,548, *p* = 0.014).

### Seasonal Training Workload

Training load characterization of the 4 weeks before the last three assessment points is presented in Table 2. These results point out that M3 was the assessment point preceded by the 4 week period with the heaviest training load, both in volume and intensity, which is intended to stimulate adaptation, and that M4 was preceded by a reduction of training load (taper) in order to allow for recovery and potentiate performance at competition.

**TABLE 2:** Mean ± SD values of the weekly training volume (m), weighed volume (m), and load score (AUL), performed every 4 weeks before the last three assessment points (M2, M3, and M4).

Training load parameters and training zones of intensity (weekly values)	M2	M3	M4	Statistic	
				*F*	*p*
Volume (m)	30,979 ± 4,120	47,251 ± 12,819**	30,110 ± 7,519***	112.668	<0.01
Weighed volume (m)	73,956 ± 10,456	112,344 ± 28,542**	73,121 ± 16,586***	144.335	<0.01
Load score (AUL)	12.1 ± 0.6	13.9 ± 0.2**	11.5 ± 0.7**;***	214.309	<0.01

AUL, arbitrary units of load; ** Different from M2; *** Different from M3 (p < 0.05).

### Immune Changes in Response to the Swimming Training Season

Regarding the effects of sex, swimming age group and maturity, no influence was observed for maturity Tanner stages on the response of the variables of interest to the training season. However, sex influenced the response of monocytes [*F*(2.931, ∞) = 3.598; *p* = 0.014], and swimming age group influenced the response of monocytes [*F*(5.271, ∞) = 2.574; *p* = 0.022], total lymphocytes [*F*(4.967, ∞) = 3.043; *p* = 0.010], and lymphocytes subsets CD3^+^ [*F*(4.678, ∞) = 2.857; *p* = 0.016], and CD4^+^ [*F*(4.550, ∞) = 2.493; *p* = 0.034] to training.

CD19^+^ lymphocytes revealed higher values for males than females throughout the season [*F*(51.314) = 4.635; *p* = 0.036] although they presented similar responses to the training season.

**FIGURE 1:**
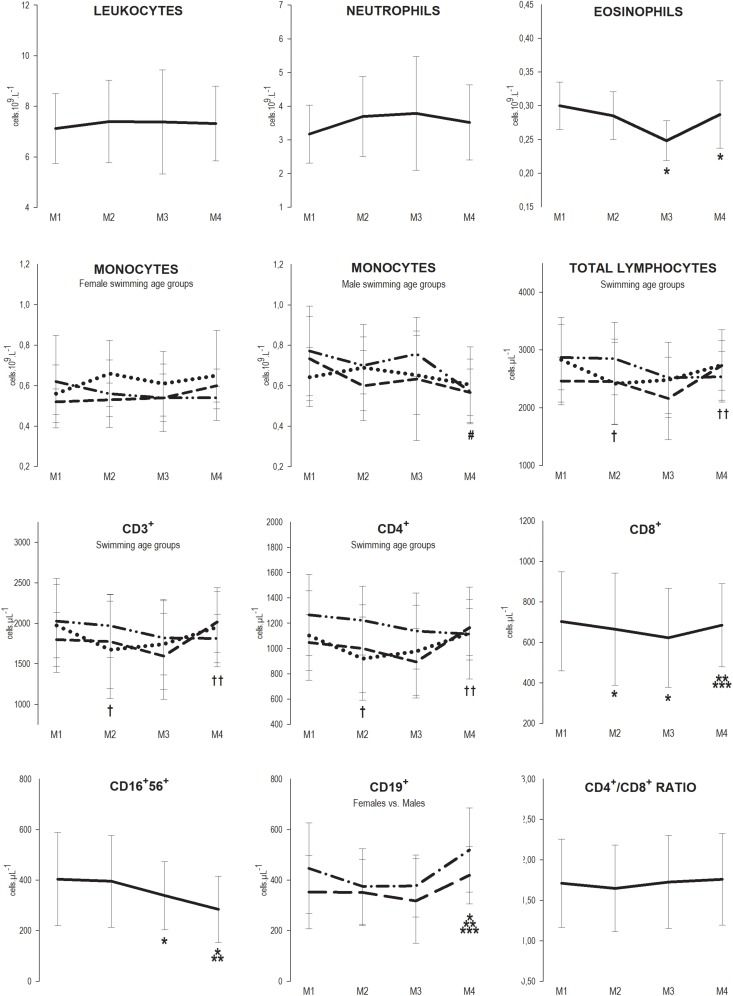
Mean ± SD values of leukocytes, neutrophils, eosinophils, total lymphocytes and subsets CD3^+^, CD4^+^, CD8^+^, CD16^+^56^+^, CD19^+^ counts, and CD4^+^/CD8^+^ ratio, at the four assessment points of the 7-month winter swimming training season (30 weeks). M1 = beginning of the season (1st week), M2 = after the main competition of the 1st macrocycle (13th week), M3 = preparatory phase of the 2nd macrocycle (23rd week) and M4 = after the main competition of the 2nd macrocycle (30th week). Legend: —, * Whole Group; ⋅⋅, † Youth; - -, ‡ Juniors; — - - —, # Seniors; — — Females; — - — Males; *, †, # different from M1; **, †† different from M2; *** different from M3 (*p* < 0.05).

Regarding the effect of training, we observed that at M2, the CD8^+^ subset decreased 5.4% (M1 = 703 ± 245 vs. M2 = 665 ± 278 cell μL^−1^; *p* = 0.032), and total lymphocytes and subsets CD3^+^ and CD4^+^ also decreased 14.6, 15.3, and 15.7%, respectively (TL, M1 = 2831 ± 734 vs. M2 = 2417 ± 714 cell μL^−1^; *p* = 0.007, CD3^+^ M1 = 1974 ± 581 vs. M2 = 1672 ± 603 cell μL^−1^; *p* = 0.003, and CD4^+^ M1 = 1102 ± 353 vs. M2 = 929 ± 329 cell μL^−1^; *p* = 0.002) but only in youth. At M3, the CD8^+^ subset remained below baseline values (M3 = 622 ± 245 cell μL^−1^; *p* = 0.008), and eosinophils and the CD16^+^56^+^ subset decreased 16.7, and 15.9%, respectively (eosinophils M1 = 0.30 ± 0.04 vs. M3 = 0.25 ± 0.03 10^9^ L^–1^; *p* = 0.003; CD16^+^56^+^ M1 = 403 ± 184 vs. M3 = 339 ± 135 cell μL^−1^; *p* = 0.019); in youth, total lymphocytes and subsets CD3^+^ and CD4^+^ recovered to baseline values but not in juniors or seniors. At M4, the CD19^+^ subsets were elevated 17.4% (M1 = 403 ± 170 vs. M4 = 473 ± 151 cell μL^−1^; *p* = 0.022), the CD16^+^56^+^ subset continued to decrease 16.2% (M4 = 284 ± 131 cell μL^−1^; *p* < 0.001), eosinophils remained below baseline levels (M4 = 0.29 ± 0.05 10^9^ L^–1^; *p* = 0.002) and the CD8^+^ subset recovered to baseline levels; monocytes also decreased 26.0% in male seniors (M1 = 0.77 ± 0.22 vs. M4 = 0.57 ± 0.16 10^9^ L^–1^; *p* = 0.031) (Figure 1).

### Upper Respiratory Symptoms

**FIGURE 2:**
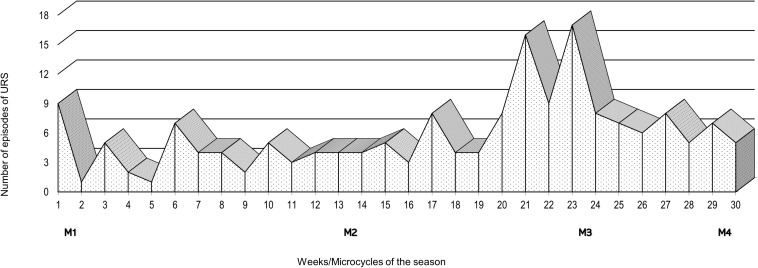
Weekly number of episodes of Upper Respiratory Symptoms (URS) over the course of a 7-month swimming winter training season and schedule of the four assessment points: M1, M2, M3 and M4.

The number of episodes of URS was monitored weekly throughout the 7-month swimming winter training season (Figure 2). An increase in the number of URS episodes was evident during the 4 weeks prior to M3, and in the 2 weeks after M3.

## Discussion

The number of studies assessing the effect of long-term training periods on the chronic response of leukocytes and subsets (including lymphocytes subsets) in athletes of different sports such as swimming (Gleeson et al., 1995; Mujika et al., 1996; Morgado et al., 2012, 2017; Rama et al., 2013; Teixeira et al., 2014), running (Denguezli et al., 2008), basketball (Brunelli et al., 2014), volleyball (Dias et al., 2011), and soccer (Suda et al., 2013; Del Giacco et al., 2014) is scarce, especially if we consider articles that assess the incidence of URS. Due to the differences in competitive schedule and training season organization, comparison of patterns of adaptations of the immune system to long-term training between different sports can be misleading. When swimming training is concerned, the length and seasonality of the training programs are rather consistent between squads and coaches. Therefore, since the swimming training season is particularly different from other sports, we chose to focus our discussion on previous studies that monitored immunological alterations along swimming training seasons.

Bearing in mind these considerations, most studies have mainly reported immune adaptations to long-term swimming in male and adult swimmers (Gleeson et al., 1995, 2000; Mujika et al., 1996; Morgado et al., 2012; Rama et al., 2013; Teixeira et al., 2014). Our study used an ecological approach where a large number of athletes was organized according to sex, maturity and swimming age group, allowing for the characterization and discrimination of the specific immune patterns of adaptations along two macrocycles of a swimming season. Additionally, the adaptations of the immune cell counts were associated with indicators of training load and athletes’ health along the training season.

Our study shows that although the mean values of the number of circulating immune cells were within the clinically normal reference range, training periods with higher load induced a decrease of the number of some of the subsets of these cells that seems to be coincident with a greater incidence of URS episodes (Figure 2). We were also able to observe that innate and acquired immune cells display different patterns along the swimming season, with innate immunity (e.g., CD16^+^56^+^) showing a cumulative effect toward the decrease of the number of cells, while the acquired immunity (e.g., CD8^+^) appears to be more affected at the most intense phase but recover after taper. Furthermore, our younger swimmers presented reduced acquired immune cells numbers (e.g., TL, CD3^+^, and CD4^+^) earlier in the training season.

Regarding the patterns of circulating cell, the lower cell counts of CD16^+^56^+^ values observed at M3 in the present study have also been reported by Rama et al. (2013) at the heaviest training period of evaluation of their study. However, the lower values we observed for the CD8^+^ subsets at M2 and M3 were not verified for these cells throughout the several assessment points by Teixeira et al. (2014). Decreases of circulating numbers of immune cell are frequently observed soon after intense, prolonged exercise and have been interpreted as an impairment of the cellular immunity – the open window theory (Walsh et al., 2011). More recently Campbell and Turner (2019) have argued that this decrease does not necessarily implicate an increased risk of infection as it results from the redistribution of immune cells from the blood compartment into more vulnerable tissues, increasing protection. Additionally, some evidence suggests that these cells may become more competent in response to exercise (Campbell and Turner, 2019). Furthermore, the entrance of these cells into the tissues may promote their apoptosis and removal from circulation inducing an increased production by the bone marrow and/or release by the lymph reservoirs and consequently the renewal of the circulating immune cells. However, the effect of frequent renewal, as expected in athletes with regular training, is still not well understood, and whether increased or decreased immune competence occurs still needs further investigation. This is particularly relevant in athletes where energy and essential nutrients can be limited due to very high loads of training or to food ingestion restrictions associated to the need of body composition management.

Although the variations of the immune cells observed along the training season kept the numbers within the clinically normal reference range, not anticipating or suggesting an impairment of the immune defenses or an increased susceptibility to viral infections, and we did not evaluate any functional parameter of these cells that may suggest increased or decreased immune competence, in our study the decreases in eosinophils, and CD16^+^56^+^ and CD8^+^ subsets were concomitant with the increase of the number of URS (M3). Thus, more controlled studies should be performed in other to understand whether or not these decreases, observed at rest after at least 12 h of exercise practice, can in fact increase the risk of URS at periods of very high load.

The elevated number of the CD19^+^ subsets observed at the end of the season compared with baseline but within the clinically normal reference range, is also discordant from the unaffected response to training of these cells reported by Gleeson et al. (1995). The CD19^+^ subsets were also higher in males than in females throughout the season, although the response to training was similar. Theoretically, these higher CD19^+^ levels confer the capacity to produce more antibodies, thus increasing humoral immunity and consequently bacterial and viral defenses. As in our study, other authors reported stable values of leukocytes (Gleeson et al., 1995; Mujika et al., 1996) and neutrophils (Mujika et al., 1996; Morgado et al., 2012).

When analyzing the variations of immune parameters, one should take into account that blood is one of the major streams for spreading the immune agents throughout the body. Plasma volume alterations along the sport season have been often reported, in particular plasma volume increases (Selby and Eichner, 1994; Kargotich et al., 1998). This hypervolemia can be an advantage as it usually results from blood volume expansion that can benefit athletic performance by increasing stroke volume, improving thermoregulatory efficiency and decreasing blood viscosity. Despite this, it is temporary and disappears within a few days of training interruption (Selby and Eichner, 1994) and can be solely explained by the increase in plasma due to compartment extravasation, without red blood cells exchange (Convertino, 1991). But from 4 weeks to 4 months, as long as the training protocol is maintained, increases in blood volume are due to both plasma volume and red blood cells mass increases. One potential explanation for plasma volume expansion is related to the increase of circulating proteins (Convertino, 1991). These proteins seem to originate mainly from the lymphatic system reservoirs, or to be due to a shift from the intracellular and interstitial space to the intravascular space, or even to *de novo* protein synthesis.

Thus, not only the plasma volume variation may influence the interpretation of the immune system parameters, but it also may result in part from the response of the immune system to a periodic stress such as the one originated from the training programs. Considering the observed plasma volume variations from M1 to M2, M3, or M4, we can argue that they cannot justify the variation of the immune parameters, on the contrary, they tend to minimize the decreases observed from M1 to M2 and the increases observed from M2 to M3 or M4. We should also consider that the effects of prolonged, intense exercise on the immune system may last for more than 24 h (Morgado et al., 2016) and that regular training, with sessions less than 24 h apart, may not allow for full recovery.

Considering the maturity status, immune cells counts values throughout the training season were similar between adolescents and adults as classified by Tanner (1962). The immune system is highly influenced by the physiological levels of some hormones (e.g., growth hormone, cortisol, estrogen, and testosterone) that are permanently changing during puberty (Gleeson, 2006). However, we have to address that the observation that no influence of maturity was observed on the immune cells numbers throughout the season might have been due to the methodology used to assess maturity stage. This methodology does not allow for the positioning of swimmers in a continuous distribution and it is a self-reported methodology with a considerable level of subjectivity. Additionally, it does not account for psychological maturity, which may be crucial to determine the athlete’s involvement in the training process. Although not supported by any evaluation of biological or psychological maturity, swimmers are in practice classified according with swimming age groups using chronological age ranges that differ between sexes. This difference aims to take into consideration the classical earlier maturational development of girls compared to boys that occurs throughout adolescence (Boggin, 1999) and imposes different training programs, specially between the youth group and the other two groups who train more similarly. In fact, in this study, the swimming age group classification of participants revealed more differences in the evolution of the immune cell counts throughout the training season than Tanner’s stages of maturity. Thus, in the youth group total lymphocytes and subsets CD3^+^ and CD4^+^ decreased at M2 and recovered to baseline values afterward. This result suggests that the initial training load of the season affected the counts of the acquired immune parameters, in particular CD4^+^ (T *helper*), with reflections on CD3^+^ (total T) and even on total lymphocytes specifically in the youth group. This behavior was not expected hence the most intense period of training was M3. In fact, juniors and seniors showed total lymphocytes and subsets CD3^+^ and CD4^+^ counts similar to baseline throughout the season, which is in accordance with previous studies that evaluated primarily junior and senior swimmers (Gleeson et al., 1995; Mujika et al., 1996; Teixeira et al., 2014). However, this difference in the youth group may be related with the traditional steeper increase in the training load that characterizes the transition for this age group.

Furthermore, males presented higher monocytes counts than females throughout the season, and the response to training was different between males and females. Male seniors showed diminished monocytes count at M4 compared to M1 and male juniors showed a similar trend profile. Although this last assessment point was preceded by a taper period, both seniors and juniors had the lower monocytes count, suggesting a cumulative effect of the training load, from which swimmers could not efficiently recover even after taper. This cumulative effect was also noticed for the CD16^+^56^+^ subset and eosinophils in the whole group but not for the CD8^+^ or CD19^+^ subsets, which recovered at the end of the season, with CD19^+^ even increasing.

Increases in training load in well-trained athletes undertaking a period of intensified training such as M3 have been described as causing the reduction of circulating immune cells counts. In our study, the reduction of the immune cell number was more evident for the innate immunity that decreased during the heaviest training period and persisted below baseline levels until the end of the season although the training load decreased. A decrease of the immune cell numbers was also noticed for the acquired immunity but earlier in the training season, suggesting a higher susceptibility to the cumulative training load of the innate immunity while acquired immunity seems to be able to adapt and recover more efficiently when the swimmer is allowed a period of taper. Furthermore, T and B lymphocytes functions have been shown to be sensitive to increases in the training load in well-trained athletes, with falls in circulating type 1 T cells counts, decreased T cell proliferative responses and reductions in stimulated B cell immunoglobulins synthesis (Verde et al., 1992; Baj et al., 1994; Lancaster et al., 2004). The cause of this depression in acquired immunity may be related to the cumulative effects of repeated bouts of intense exercise which can cause elevations of the circulating stress hormones, particularly cortisol, and anti-inflammatory cytokines (e.g., IL-6, IL-10, IL-Ira) (Gleeson and Bishop, 2005). Overall, the result appears to be a temporary inhibition of Type 1 T cell cytokine production, with a relative diminution of the Type 1 (cell-mediated) response (Gleeson and Bishop, 2005). The literature refers cortisol as a potential conditioner of the entry of lymphocytes into the circulation after intense and prolonged exercise contributing to their return to lymphoid compartments (Nieman, 1994). However, the overall long-term training effects of cortisol over lymphocytes remain unclear. An augmented incidence of viral infections can be the consequence of a defect in T cell number and function (Fabbri et al., 2003) either associated or not with cortisol action.

At the heaviest training period both innate and acquired immune cells counts decreased and a higher frequency of self-reported URS episodes was evident during this training phase. Although, as stated before, one cannot assume the reduction of immune cells counts implies the reduction of the immune function, this outcome may reinforce the idea of a disturbed immune resilience of the swimmers. Our results are in agreement with other studies that have also reported an increase in URS symptoms during the heaviest training periods characterized by high loads imposed continuously over several weeks (Morgado et al., 2012; Rama et al., 2013).

However, we would like to reinforce that despite the steep changes in the number of immune cells, all the observed mean values were within the clinically normal reference range values. In addition, as the different types of cells have redundant actions, one should argue whether small reductions of the numbers of some of them are effectively relevant for the overall immune protection. A broader panel of immune parameters, including indicators of cell activity, would have been useful to address this issue but we have focused on the immune cells counts typically evaluated in haemogram and leucograms used for clinical purposes and thus our conclusions cannot be extended to mechanistic hypothesis.

In addition, the timing of blood sampling does not rule out that the immune cell counts had not recovered to pre-exercise values. However, our ecological approach was limited by our goal to perform an observational study without interfering with the training process. All participants were evaluated in the morning in order to eliminate circadian variations due to cortisol cycles. As high-level swimming training requires that athletes train from Monday to Saturday and sometimes twice a day regularly, a limited number of athletes had to be evaluated after having trained in the previous afternoon (low intensity session). Balancing this limitation, it gives us a realistic evaluation of the immune cell counts of the athlete in the morning of a regular training day.

Bearing in mind these limitations, the results of the present investigation enhance the importance of controlling immunological alterations during in-season training to prevent the decrease of the numbers of the innate and acquired immunity and consequently the higher incidence of URS which may compromise the attendance to training sessions and the improvement of performance. This is particularly important in heavy training periods but also in the first months of training for young athletes when the workload increase is steep. However, the training effect is not expected to alter the immune cells numbers out of the clinical normal range. Individual panels of immune parameters along the season should be studied. Further studies should be performed to understand the accumulated effect of years of training on the immune system.

## Conclusion

The long-term swimming training season studied induced a diversified pattern of immune adaptation, with a cumulative effect toward a decrease of the number of the innate immune cells (e.g., CD16^+^56^+^), while the number of acquired immune cells (e.g., CD8^+^) appeared to be more affected at the most intense training phase, recovering after a taper. Younger swimmers presented reduced acquired (e.g., TL, CD3^+^, and CD4^+^) immune cells numbers earlier in the training season. The innate and acquired immune cells numbers reduction that occurred at the heaviest training period might have contributed to an immune perturbation manifested as a higher prevalence of upper respiratory symptoms.

## Data Availability Statement

The datasets generated for this study are available on request to the corresponding author.

## Ethics Statement

The studies involving human participants were reviewed and approved by Ethics Committee of the Faculty of Human Kinetics of the University of Lisbon. Written informed consent to participate in this study was provided by the participants’ legal guardian/next of kin.

## Author Contributions

JM, CNM, FA, and CPM conceived and designed the study. JM, CNM, JR, and DC conducted experiments and analyzed the data. JM, CNM, JR, and CPM wrote the manuscript. All the authors read and approved the manuscript.

## Conflict of Interest

The authors declare that the research was conducted in the absence of any commercial or financial relationships that could be construed as a potential conflict of interest.
